# FOXP3 as a prognostic marker and therapeutic target in immunogenic cell death modulation for clear cell renal cell carcinoma

**DOI:** 10.1007/s12672-025-01831-w

**Published:** 2025-01-30

**Authors:** Jian Chen, Cheng Zhu, Yan He, Liping Huang, Weizhuo Wang, Shuaishuai Huang

**Affiliations:** 1 Medical Department, Ningbo Women and Children’s Hospital, Ningbo, Zhejiang China; 2 Department of Laboratory, Ningbo Yinzhou No.2 Hospital, No.998 Qianhe Road, Yinzhou Distrinct, Ningbo, 315100 China; 3 Center for Reproductive Medicine, The Second Affiliated Hospital of Soochow University, Suzhou, 215004 China

**Keywords:** Clear Cell Renal Cell Carcinoma, Immunogenic Cell Death, FOXP3, Prognosis, Tumor Immune Microenvironment

## Abstract

**Background:**

Clear cell renal cell carcinoma (ccRCC) remains a challenging cancer type due to its resistance to standard treatments. Immunogenic cell death (ICD) has the potential to activate anti-tumor immunity, presenting a promising avenue for ccRCC therapies.

**Methods:**

We analyzed data from GSE29609, TCGA-KIRC, and GSE159115 to identify ICD-related prognostic genes in ccRCC. By applying consensus clustering, patients were categorized based on ICD modification patterns, and an ICD signature (ICDS) model was developed using a PCA approach. Functional studies were conducted with FOXP3 knockdown in ccRCC cell lines to explore its impact on cell behavior.

**Results:**

Eleven ICD-related genes were identified as key prognostic indicators in ccRCC, with high ICDS linked to worse survival outcomes. High ICDS also correlated with increased levels of immune-suppressive cells within the tumor microenvironment. FOXP3 was highlighted as a critical gene influencing ICD, where its knockdown significantly reduced ccRCC cell proliferation and migration, underscoring its role in tumor progression.

**Conclusions:**

This study establishes FOXP3 as a pivotal factor in ICD regulation and ccRCC progression. Targeting FOXP3 and other ICD pathways could enhance treatment efficacy in ccRCC, providing a foundation for ICD-based therapeutic strategies. Evaluating ICD patterns in ccRCC may guide patient-specific interventions, paving the way for improved management of this aggressive cancer.

**Supplementary Information:**

The online version contains supplementary material available at 10.1007/s12672-025-01831-w.

## Introduction

Clear cell renal cell carcinoma (ccRCC) is the most prevalent type of renal cancer, making up 70–80% of all cases. It is marked by its high lipid and glycogen content, which gives the cells a clear appearance, and is characterized by its aggressive nature and poor prognosis, especially in advanced stages [1]. The disease's pathophysiology is driven by complex genetic and molecular changes, including VHL gene mutations, leading to dysregulation of hypoxia-inducible factors that promote angiogenesis and tumor growth [2, 3]. Despite progress in surgical and targeted therapies, ccRCC remains difficult to treat because of its inherent resistance to conventional chemotherapy and radiation [4, 5]. The tumor's heterogeneity and its ability to evade immune detection further complicate effective management, highlighting the need for new therapeutic strategies.

Immunogenic cell death (ICD) is a controlled process that triggers an adaptive immune response targeting dying cancer cells [6]. Unlike other types of cell death, ICD is distinguished by the release of damage-associated molecular patterns (DAMPs) like ATP, and HMGB1, which strongly signal dendritic cells and other antigen-presenting cells [7, 8]. These signals promote the uptake of tumor antigens and activate T cells, leading to a potent anti-tumor immune response [9]. ICD mechanisms involve various stress pathways, including endoplasmic reticulum stress and the generation of reactive oxygen species, which together initiate and propagate immunogenic signals [10, 11]. In ccRCC, exploiting ICD offers a promising approach to conquering the immunosuppressive TME and improving effectiveness of immunotherapies.

FOXP3 is a transcription factor predominantly recognized for its role in the development and function of regulatory T cells (Tregs), a key component of the immune microenvironment [12]. Tregs play a critical role in maintaining immune homeostasis and preventing autoimmunity by suppressing excessive immune responses. In the context of cancer, FOXP3 is often associated with the immunosuppressive tumor microenvironment (TME), as its expression correlates with increased Treg infiltration, leading to the inhibition of anti-tumor immunity [13]. This immunosuppressive effect facilitates tumor immune evasion, contributing to cancer progression and poor prognosis. Beyond its role in immune suppression, FOXP3 has been implicated in regulating other cellular and molecular pathways in the TME. FOXP3 modulates the expression of immune checkpoint molecules such as PD-L1 and CTLA-4, enhancing the tumor's ability to escape immune surveillance [14]. Additionally, FOXP3 interacts with key signaling pathways, including TGF-β and Wnt/β-catenin, which are critical for maintaining the immunosuppressive and pro-tumorigenic characteristics of the TME.

Additionally, identifying and validating specific ICD biomarkers could greatly improve ccRCC treatment. These biomarkers would enable better patient stratification, allowing clinicians to identify those most likely to benefit from ICD-inducing therapies. Monitoring these biomarkers could lead to more precise, tailored treatments, ultimately enhancing patient outcomes.

## Methods

### Data sources and processing

The expression profiles and clinical information were retrieved from GSE29609 [15] and TCGA-KIRC. Additionally, single-cell sequencing data for GSE159115 [19] were obtained from the TISCH2 database [16].

### Identification of prognostic ICD-related genes

We compiled 29 ICD-related genes from relevant literatures [17, 18]. Differentially expressed genes from GSE159115 were identified using a significance threshold of *P* < 0.05. The intersection of differentially expressed genes and ICD-related genes was then analyzed, with prognostic genes determined via univariate Cox regression analysis. The gene set "c2.cp.kegg.v6.2" was downloaded from the MSigDB database [20].

### Construction of the ICD prognostic model

The process for constructing the ICDS involved the following steps: First, we identified and normalized differential genes between ICD-related subtypes, focusing on overlapping genes. we applied univariate Cox regression analysis to identify genes with significant prognostic value. Principal Component Analysis (PCA) was used to develop the ICDS, specifically leveraging Principal Components 1 (PC1) and 2 (PC2) as they accounted for the majority of variance in the dataset while minimizing noise from less relevant components. These principal components were selected because they represent the most well-connected quality blocks within the dataset, capturing the primary patterns of co-expression among ICD-related prognostic genes. The formula for the ICDS was defined as: ICDS = ∑(PC1i + PC2i), where i represents the expression levels of the selected genes. This approach ensures that the ICDS reflects the aggregate impact of key ICD-related genes on patient prognosis.

### Immune suppression cell infiltration and functional pathway enrichment analysis in ccRCC

The ESTIMATE method determined the immunoscore, estimate score, and stromal score in the TME of ccRCC samples. Immune therapy data for ccRCC from TCIA were downloaded, and differences in immune therapies between groups were further analyzed.

### Somatic mutation analysis and drug sensitivity analysis in ccRCC

The "maftools" R package was utilized to visualize somatic mutation profiles and identify frequently mutated genes between different ICDS groups. TMB differences between groups were analyzed, with KM curves used to evaluate survival differences between TMB and combined ICDS. For targeted therapy drug analysis, the "prophetic" package assessed the differences in IC50 of 12 common chemotherapy drugs for renal cancer.

## Results

### Identification of prognostic genes in CCRCC through comprehensive dataset analysis

**Fig. 1:**
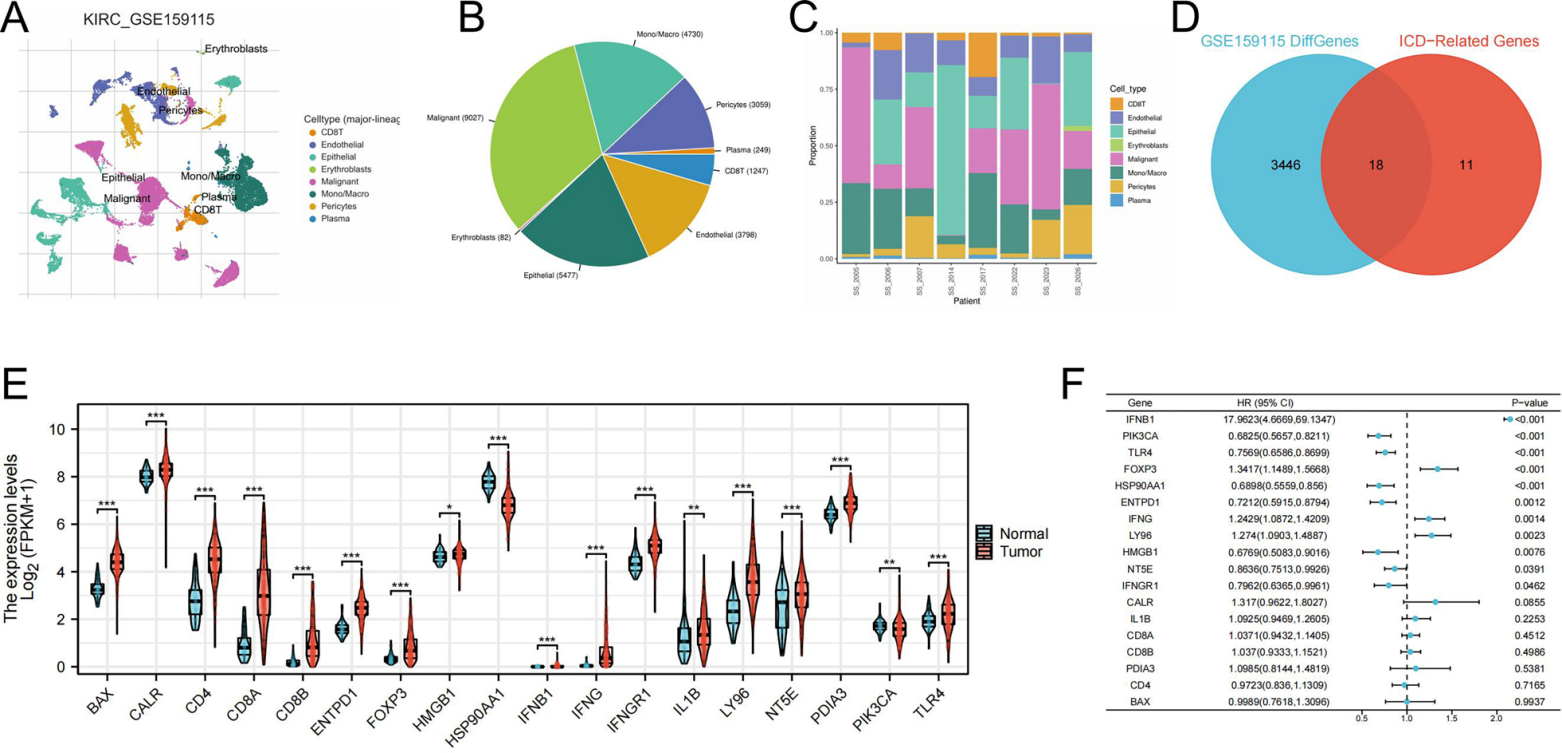
Cell Type Distribution and Gene Expression in ccRCC Samples. (**A**) Cluster analysis of cell types identified in the GSE159115 dataset, grouped into eight distinct types; (**B**) Proportional representation of these cell types across analyzed ccRCC samples; (**C**) Spatial distribution of cell types across different ccRCC samples; (**D**) Cross-analysis of ICD-related genes and differentially expressed genes identifying 18 genes of intersection; (**E**) Comparison of the expression of 18 genes of intersection between cancerous and normal tissues; (**F**) Univariate Cox regression analysis of the 18 identified ICD-related genes

We analyzed the GSE159115 dataset using the TISCH2 database, clustering it into eight distinct cell types (Fig. 1A). The proportion of these cell types in the samples and their distribution across different samples are shown in Figs. 1B and C. Subsequently, applying an adjusted p-value threshold of < 0.001, we identified 3,464 genes exhibiting significant differential expression from the GSE159115 dataset. A cross-analysis with ICD-related and differentially expressed genes revealed 18 genes of interest (Fig. 1D). Further analysis in the TCGA-KIRC dataset revealed that these 18 genes significantly differed between cancerous and normal tissues (Fig. 1E). A univariate COX regression analysis identified 11 out of the 18 ICD-related genes as being related to prognosis (Fig. 1F).

### Integration and clustering of ccRCC samples based on prognostic ICD-related genes

**Fig. 2:**
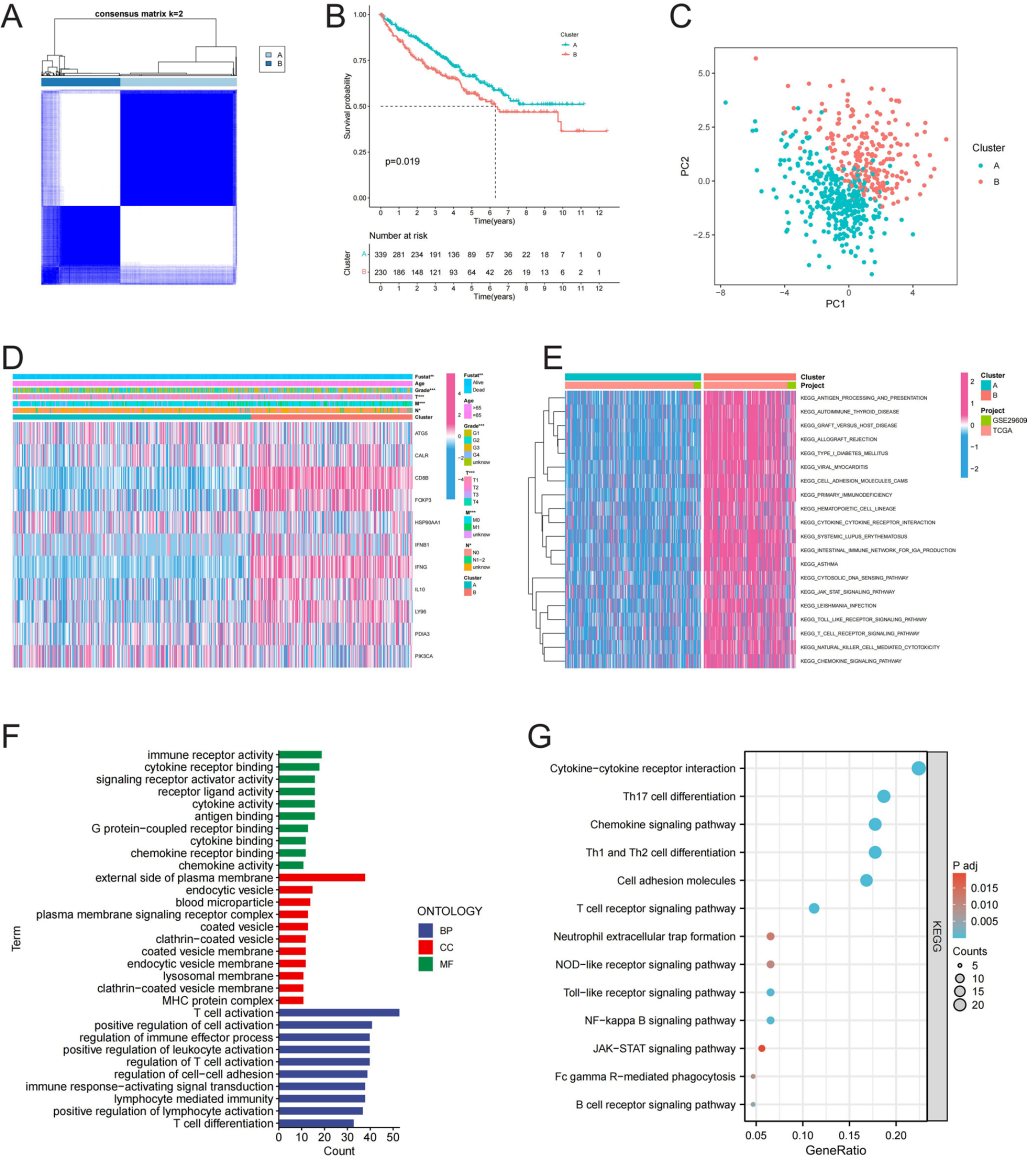
Establishment of ICD-Related Clusters. (**A**) Consensus clustering of 569 ccRCC samples based on 11 prognostic ICD-related genes; (**B**) KM curves illustrating differences in patient survival between the identified clusters; (**C**) Principal Component Analysis validating the clustering approach; (**D**) Heatmap displaying the differential expression of the 11 ICD-related genes across the clusters, with annotations for clinical variables such as grade and TNM staging; (**E–G**) GSVA and enrichment analysis highlighting differential activation of immune cell regulation and oncogenic pathways between the clusters

We merged the GSE29609 and TCGA-KIRC datasets, ultimately including 569 ccRCC samples. Based on the expression profiles of 11 prognostic ICD-related genes, we performed consensus clustering to classify patients into two distinct groups (Fig. 2A). The KM curve revealed that cluster B exhibited a worse prognosis (Fig. 2B). PCA demonstrated the effectiveness of this clustering approach (Fig. 2C). A heatmap displayed the expression of the 11 genes across the clusters, with significant differences observed in various clinical variables, including grade and TMN staging, between the two clusters (Fig. 2D). GSVA revealed significant enrichment of several oncogenic pathways in cluster B. Further GO and KEGG enrichment analyses revealed significant enrichment in immune cell regulation and oncogenic pathways (Fig. 2E-G).

### Immune infiltration differences and prognostic implications in ccRCC subtypes

**Fig. 3:**
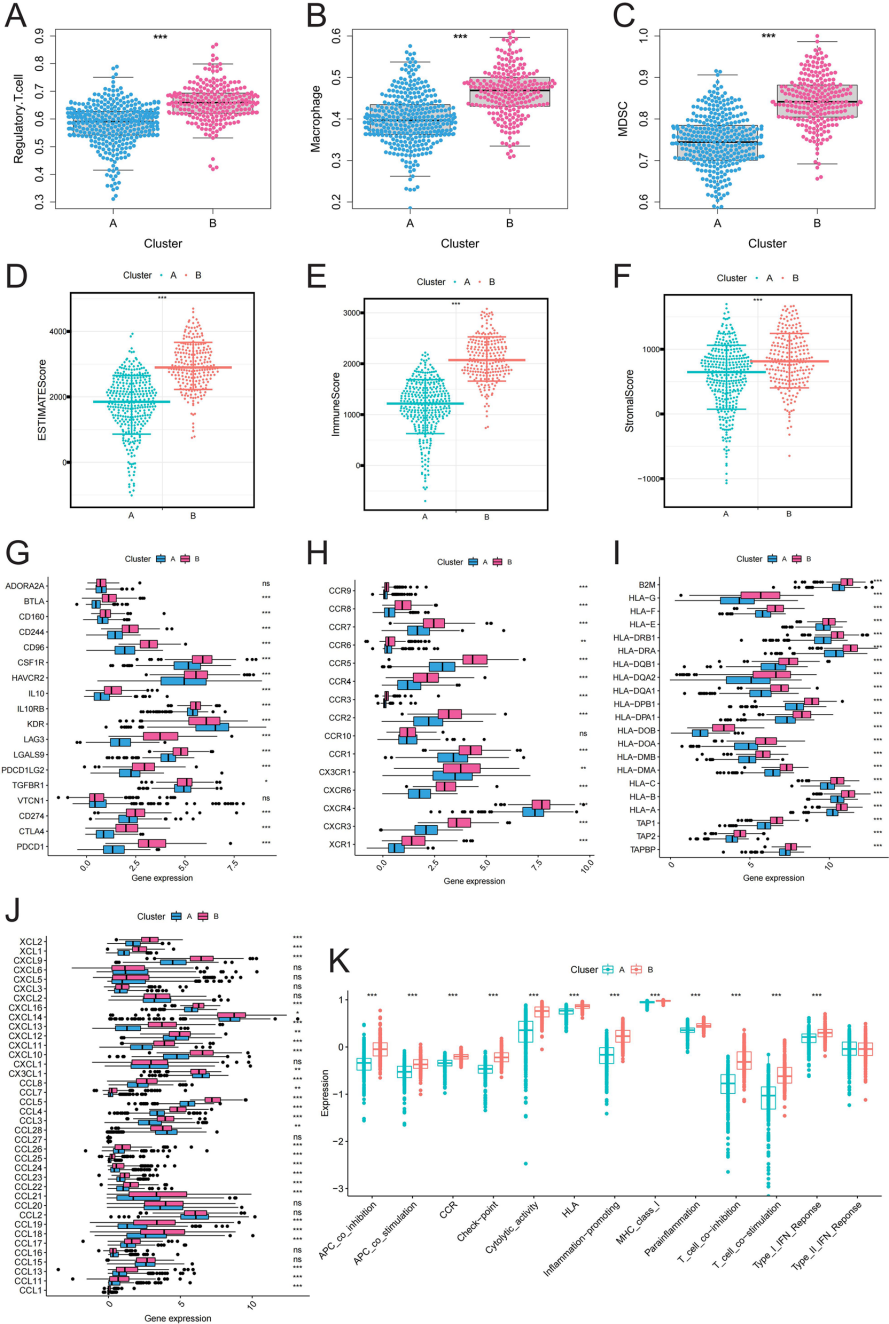
Immune Infiltration and Prognostic Implications in ccRCC Subtypes. (**A-C**) Quantification of immune suppressive cell populations (Tregs, macrophages, and MDSCs) in the identified clusters; (**D-F**) Immune, stromal, and ESTIMATE scores comparisons between the clusters; (**G-J**) Expression levels of immune checkpoint, chemokine-related, and HLA-related genes across the clusters; (**K**) Analysis of the immune function score, showing higher expression of checkpoints, HLA, and inflammation-promoting factors in the poorer prognosis cluster

To further investigate the reasons behind the prognostic differences between the clusters, we assessed the variations in immune infiltration. We observed that immune suppressive cells, like Tregs, macrophages, and myeloid derived suppressor cells (MDSCs), were significantly more abundant in cluster B (Fig. 3A-C). In the TME, cluster B exhibited significantly higher immune, stromal, and estimate scores compared to cluster A (Fig. 3D-F). Furthermore, we found that most immune checkpoint, chemokine-related, and HLA-related genes were significantly upregulated in cluster B (Fig. 3G-J). The immune function score indicated higher expression of checkpoints, HLA, and inflammation-promoting factors in cluster B (Fig. 3K). These findings suggest that the poorer prognosis observed in cluster B may be attributed to higher levels of immune suppression and activation of oncogenic pathways. This underscores the vital role of the immune microenvironment in ccRCC progression and treatment response.

### Genetic subtyping based on differential gene expression in ccRCC

The consensus clustering was employed to further classify the samples into geneclusters A and B (Fig. S1A). A heatmap displayed the gene expression patterns and the distribution of clinical variables across the geneclusters (Fig. S1B). KM survival curves indicated that patients in genecluster B had a poorer prognosis (Fig. S1C). Additionally, most of the 11 prognostic ICD-related genes showed differential expression between the geneclusters (Fig. S1D). These results, along with our previous results, highlight the essential role of the immune microenvironment and gene clusters in understanding the prognosis and progression of ccRCC. Genetic subtyping provides a more detailed molecular characterization of the disease, which can aid in identifying specific prognostic markers and potential therapeutic targets.

### Evaluation of ICD modification patterns and prognostic implications in ccRCC

**Fig. 4:**
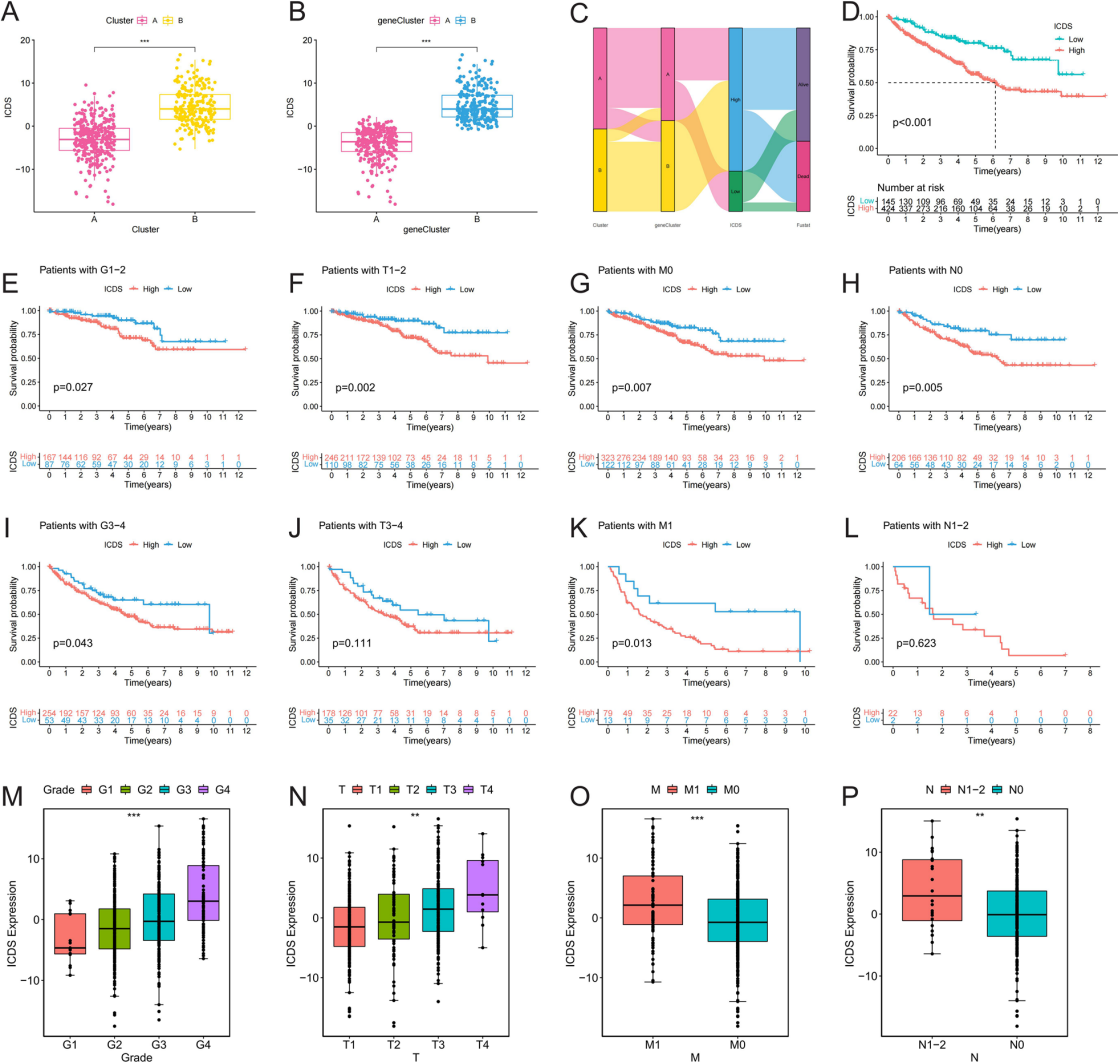
Evaluation of ICD Modification Patterns and Prognostic Implications in ccRCC. (**A, B**) Expression levels of the ICD Signature (ICDS) in ccRCC subtypes and genetic subtypes; (**C**) Alluvial diagram depicting changes in patient attributes over the course of the study; (**D**) KM curve showing the survival impact of high ICDS; (**E-P**) Analysis of ICDS expression and its prognostic implications across different clinical stages

We constructed an ICDS prognostic model using the PCA algorithm. We found that ICDS was highly expressed in both subtype B and genetic subtype B (Fig. 4A and B). Figure 4C showed an alluvial diagram that depicts the distribution of patients across different clusters, gene clusters, and ICDS groups, and their corresponding survival outcomes. It highlights the alignment between these categories, emphasizing their relevance in predicting patient prognosis. KM curve showed patients with high ICDS had a poorer prognosis (Fig. 4D). To thoroughly assess the clinical significance of ICDS, we examined its expression and prognostic differences across different clinicopathological stages. The analysis revealed that patients with high ICDS consistently exhibited poorer prognoses at various stages (Fig. 4E-L). Additionally, ICDS expression was markedly higher in advanced clinical stages (Fig. 4M-P). These findings highlight that high ICDS is associated with poorer outcomes and advanced disease stages, emphasizing the importance of considering ICD modification patterns in the clinical management of ccRCC.

### Mutational and immune characteristics of ICD prognostic model

**Fig. 5:**
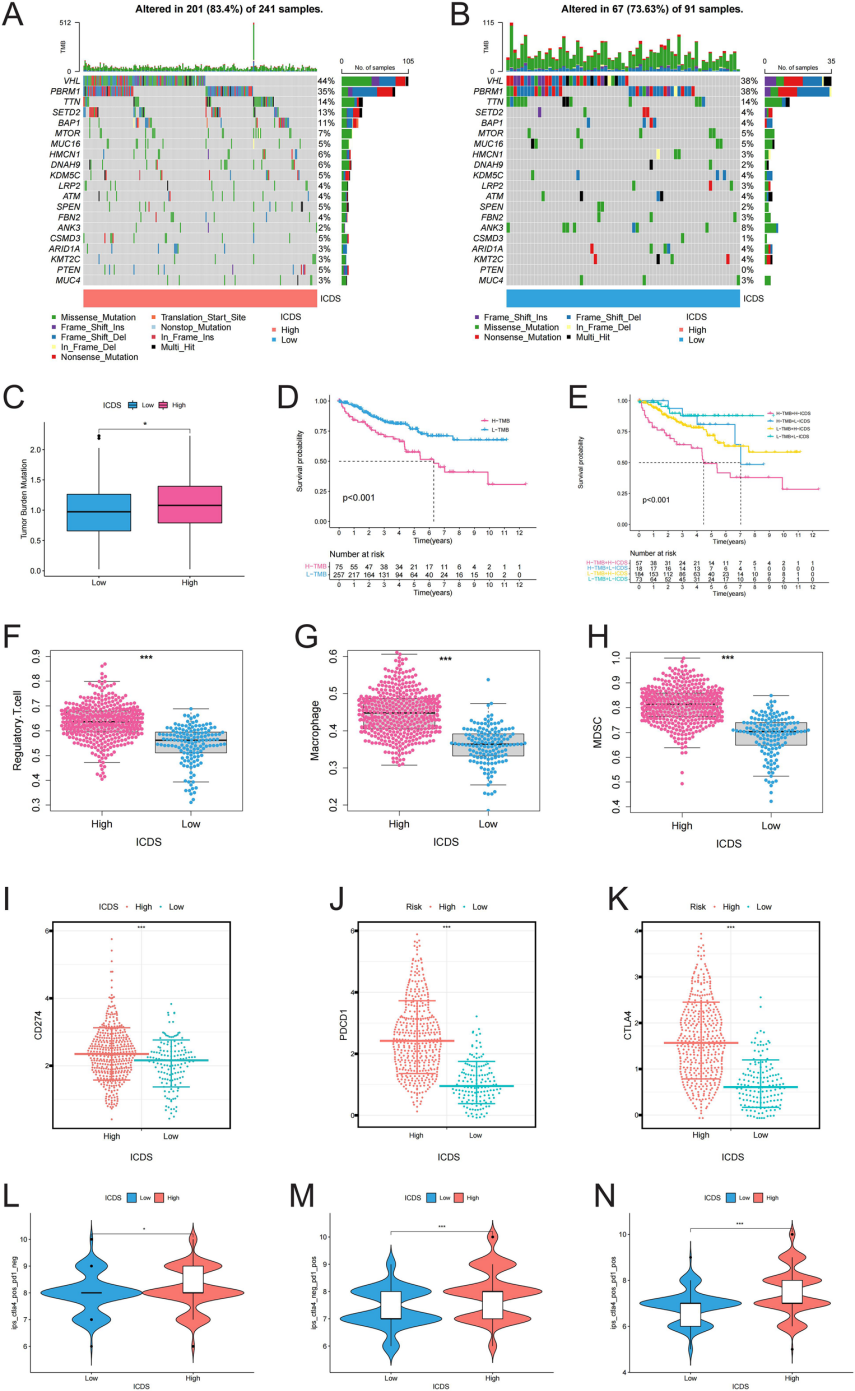
Mutational and Immune Characteristics of ICD Prognostic Model. (**A, B**) Mutation frequencies in ICDS groups, focusing on the top 20 most frequently mutated genes; (**C**) Comparison of TMB between high and low ICDS group; D: KM survival curve illustrating the impact of high TMB on patient prognosis; (**E**) Combined analysis of mutation data and survival rates, confirming the prognostic accuracy of high TMB combined with high ICDS; (**F-K**) Expression levels of immune suppressive cells and immune checkpoint molecules in the ICDS groups, highlighting their upregulation in the high ICDS groups; (**L-N**) Effectiveness of anti-PD-1 and anti-CTLA4 therapies in ccRCC patients, stratified by ICDS expression

The waterfall plot revealed a mutation rate of 83.4% in high ICDS group and 73.63% in low ICDS group, with top 20 most frequently mutated genes being consistent across both groups (Fig. 5A and B). The high ICDS group exhibited an elevated TMB (Fig. 5C). Figure 5D demonstrated that high TMB (H-TMB) correlated with a poorer prognosis. Further analysis combining mutation and survival data showed that patients with both high TMB and high ICDS (H-TMB + H-ICDS) had the poorest prognosis, reinforcing the accuracy of the previous findings (Fig. 5E). High TMB can increase tumor immunogenicity, triggering a stronger immune response, which in turn leads to the upregulation of immune checkpoint molecules by tumor cells to evade immune system attacks. We found that immune suppressive cells and immune checkpoint molecules were significantly upregulated in the high ICDS group (Fig. 5F-K). Anti-PD-1 and/or anti-CTLA-4 therapies were found to be more suitable for patients with high ICDS (Fig. 5L-N). These findings suggested that the high ICDS group was characterized by a higher mutation rate and increased expression of immune suppressive cells and checkpoint molecules, contributing to poorer prognosis. Moreover, by comparing the differences in drug IC50 between groups, we found Axitinib, Bosutinib, Gefitinib, Lenalidomide, Metformin, Nilotinib, Pazopanib, Sunitinib, Temsirolimus, Tipifarnib, Vorinostat is more suitable for patients with high ICDS (Fig. S2A-L).

### Key gene analysis in ICD modification for ccRCC

**Fig. 6:**
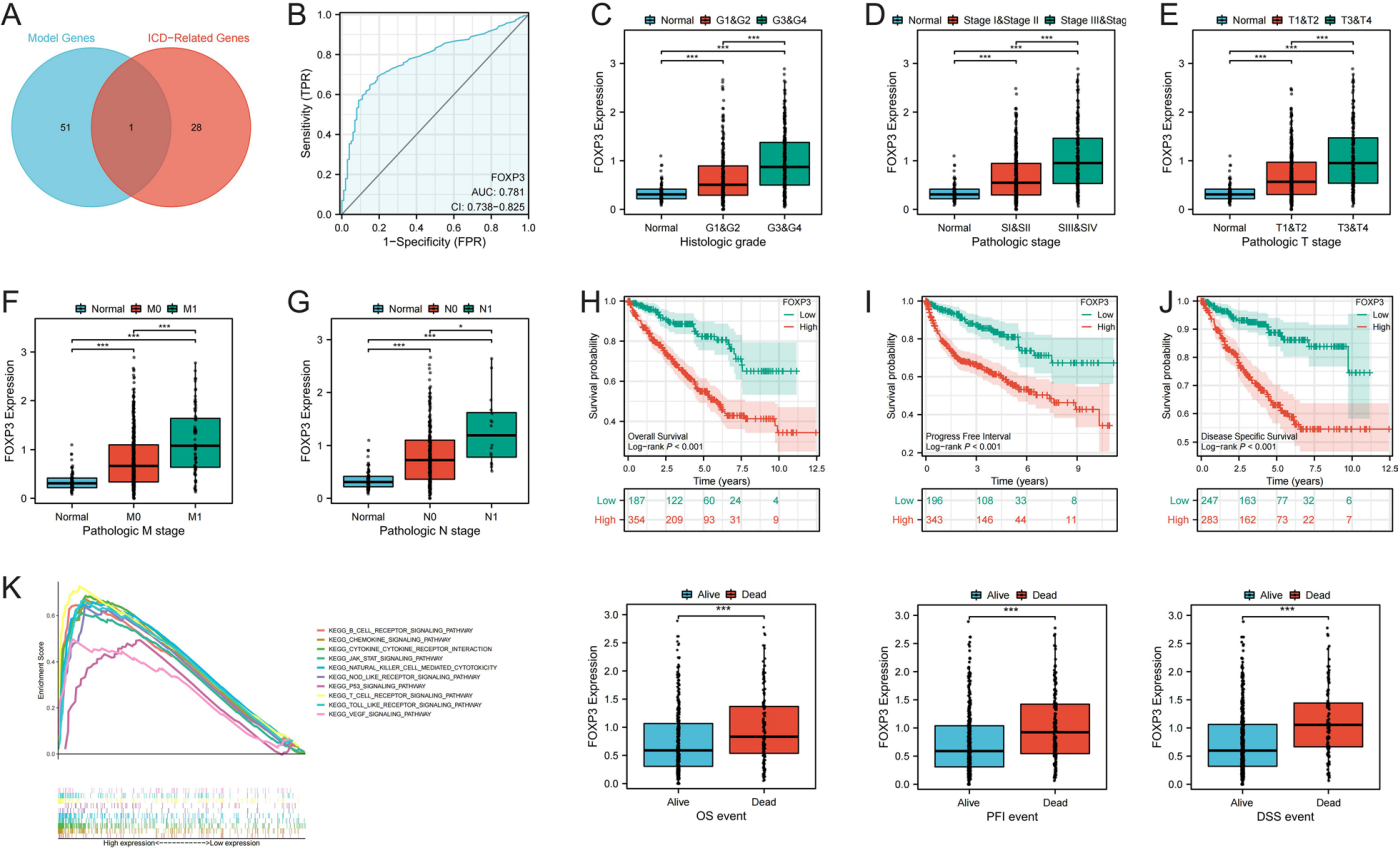
Key Gene Analysis in ICD Modification for ccRCC. (**A**) Identification of FOXP3 as the overlapping gene within the ICDS prognostic model and ICD-related genes; (**B**) Diagnostic ROC curve demonstrating FOXP3's accuracy in distinguishing between cancerous and normal tissues; (**C-G**) Expression levels of FOXP3 across different clinical stages and its correlation with advanced disease; (**H-J**) KM curves for OS, PFI, and DSS stratified by FOXP3 expression levels; (**K**) GSEA enrichment analysis of oncogenic pathways in groups with high FOXP3 expression

Given the crucial role of ICD in ccRCC, we further analyzed key genes within ICD modification. By intersecting the modeling genes used to construct the ICDS prognosis model with ICD-related genes, we identified FOXP3 as the sole overlapping gene (Fig. 6A). We then explored prognostic value of FOXP3 in ccRCC. Diagnostic ROC curve indicated that FOXP3 has good accuracy in distinguishing between cancer and adjacent normal tissues, with an AUC of 0.781 (Fig. 6B). Additionally, FOXP3 expression was markedly elevated in advanced clinical stages (Fig. 6C-G). The KM curve showed that high ICDS generally experienced poorer overall survival (OS), progression-free interval (PFI), and disease-specific survival (DSS). Furthermore, FOXP3 expression was higher in patients who had passed away (Fig. 6H-J). GSEA enrichment analysis revealed significant enrichment of several oncogenic pathways in high FOXP3 expression group (Fig. 6K). These findings highlight FOXP3 as a critical gene in ICD modification, with significant prognostic value in ccRCC.

### Immunological characteristics of FOXP3 in ccRCC

**Fig. 7:**
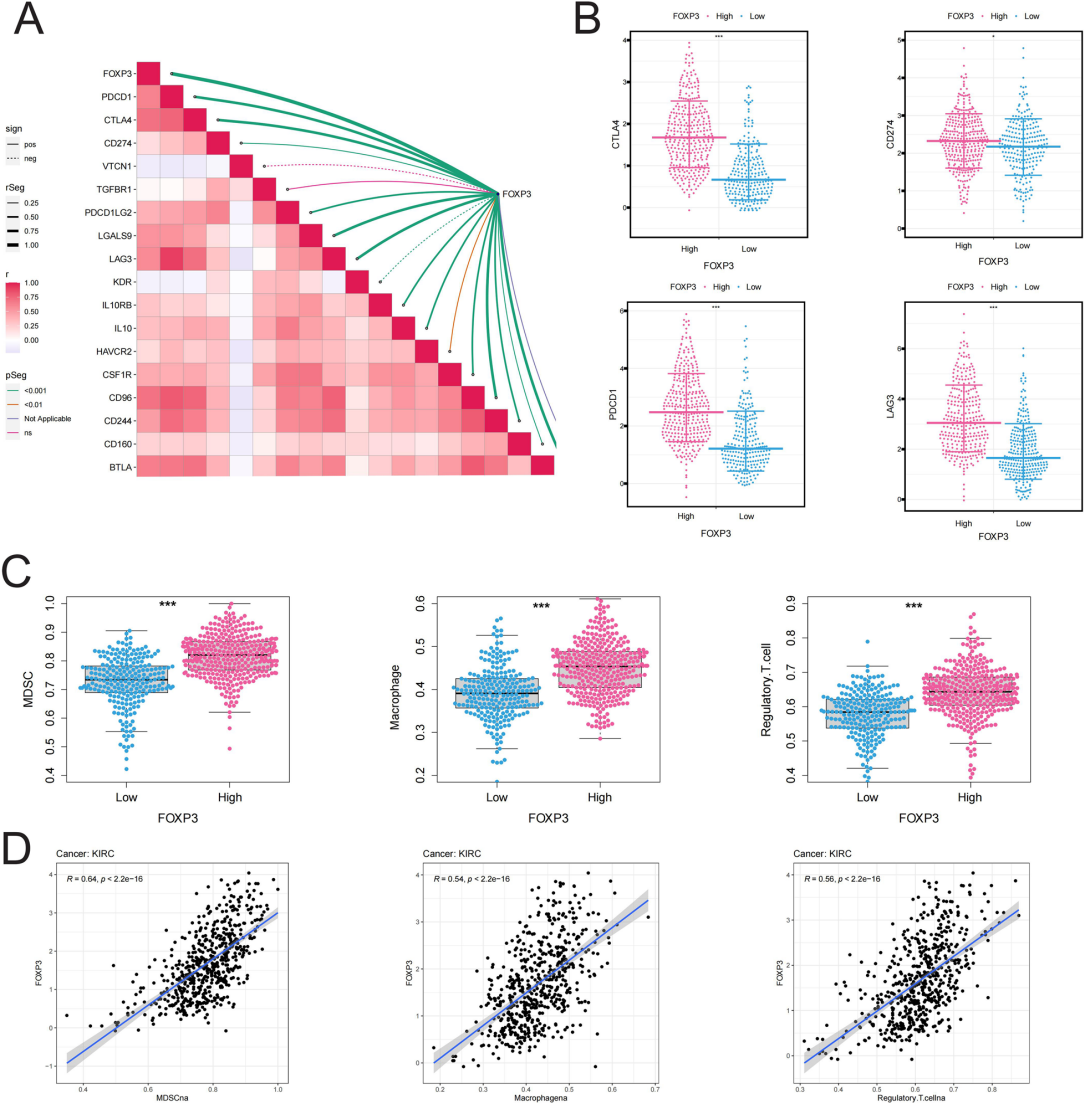
Immunological Characteristics of FOXP3 in ccRCC. (**A**) Correlation analysis showing significant positive relationships between FOXP3 and major immune checkpoint inhibitors; (**B**) Expression analysis of immune checkpoints in high ICDS group; (**C, D**) Analysis of immune suppressive cells' expression and their correlation with ICDS, demonstrating the critical role of FOXP3 in the immunosuppressive environment

We further analyzed the immunological characteristics of FOXP3 in ccRCC. We found a strong positive association between FOXP3 and most immune checkpoint inhibitors (Fig. 7A). Major immunesupressive checkpoints were elevated in high ICDS group (Fig. 7B). Additionally, immune suppressive cells were also markedly elevated in high ICDS group and showed significant positive correlations with ICDS (Fig. 7C and D). These results highlight the crucial role of FOXP3 in creating the immunosuppressive environment in ccRCC. Elevated FOXP3 expression is linked to higher levels of immune checkpoint molecules and immune suppressive cells, contributing to a poorer prognosis. This highlights FOXP3's potential as a biomarker for predicting the efficacy of immunotherapy in ccRCC patients.

### Exploring the role of FOXP3 in ccRCC cell lines

**Fig. 8:**
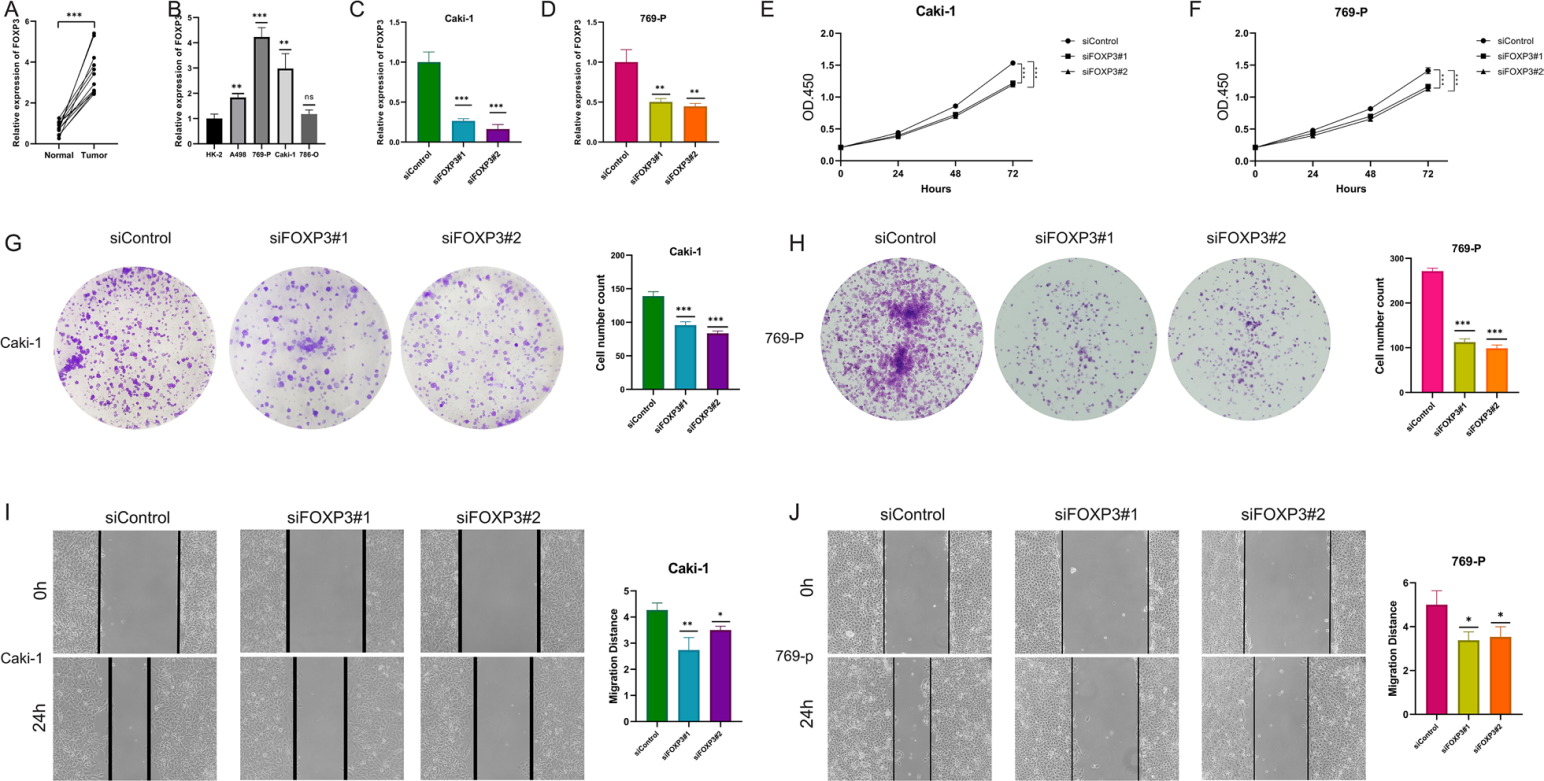
Role of FOXP3 in ccRCC Cell Lines. (**A, B**) mRNA expression levels of FOXP3 in ccRCC tissues and cell lines compared to normal tissues and HK-2 cell line; (**C, D**) Effects of FOXP3 knockdown by siRNA on mRNA levels in Caki-1 and 769-P cells; (**E, F**) CCK8 assay results showing reduced proliferative capacity following FOXP3 silencing in ccRCC cell lines; (**G, H**) Colony formation assay results showing reduced proliferative capacity following FOXP3 silencing in ccRCC cell lines; (**I, J**) Scratch assay results assessing the migration capacity of ccRCC cells post FOXP3 knockdown, illustrating reduced invasiveness

We examined expression patterns and functional roles of FOXP3 in ccRCC cell lines through a series of experiments. Figure 8A and B illustrated the mRNA expression levels of FOXP3 in ccRCC tissues and cell lines (A498, 769-P, Caki-1, and 786-O) compared to normal tissues and the HK-2 cell line. The data indicate that FOXP3 was elevated in ccRCC tissues and cell lines, suggesting its potential involvement in promoting ccRCC progression. FOXP3 mRNA expression was silenced using two different siRNAs (siFOXP3#1 and siFOXP3#2) in Caki-1 and 769-P cells. The results, as shown in Fig. 8C and D, there was a significant reduction in FOXP3 mRNA levels in the knockdown groups compared to the control, confirming the efficacy of the siRNA approach. The CCK8 assay results revealed that FOXP3 knockdown significantly impaired the proliferation ability of both Caki-1 and 769-P cells (Fig. 8E and F). The colony formation assay results revealed that FOXP3 knockdown significantly impaired the colony-forming ability of both Caki-1 and 769-P cells, indicating that FOXP3 may play a critical role in ccRCC cell proliferation (Fig. 8G and H). The scratch assay results, presented in Fig. 8I and J, evaluated the migration capacity of Caki-1 and 769-P cells following FOXP3 knockdown. The migration distance was notably decreased in cells treated with siFOXP3#1 and siFOXP3#2 compared to the control, indicating that FOXP3 supports the invasive properties of ccRCC cells. These results collectively highlighted the oncogenic potential of FOXP3 in ccRCC, impacting both cell proliferation and migration.

## Discussion

ICD is gaining prominence as a pivotal mechanism in cancer therapy due to its unique ability to convert the TME from immunosuppressive to immunostimulatory [21]. Unlike conventional cell death mechanisms that often evade immune surveillance, ICD effectively stimulates an anti-tumor immune response by presenting tumor antigens in a manner that enhances dendritic cell activation and T cell priming [22]. Recent advancements in understanding the molecular pathways involved in ICD have opened new avenues for cancer treatment [23, 24]. For instance, treatments that can induce ICD—such as certain chemotherapeutics, radiotherapy, and oncolytic viruses—have shown potential in enhancing the efficacy of cancer immunotherapies, including checkpoint inhibitors and cancer vaccines [25]. These therapies work synergistically to not only kill tumor cells via direct cytotoxic effects but also to modulate the TME in a way that is conducive to immune-mediated control. Furthermore, the clinical relevance of ICD has been supported by observational studies in various cancers, where the induction of ICD correlates with improved patient outcomes. For example, in colorectal cancer, the presence of ICD markers was related to a reduced risk of metastasis and longer overall survival [26]. This correlation underscores the potential of leveraging ICD mechanisms in designing next-generation cancer therapies that not only target the tumor cells but also transform the TME to support robust and durable anti-tumor immunity. ccRCC is characterized by unique metabolic features, including dysregulated lipid metabolism and adaptation to hypoxic conditions, which significantly influence the TME. Lipid accumulation in ccRCC tumor cells has been shown to impair dendritic cell maturation and antigen presentation, thereby dampening the anti-tumor immune response [27]. ccRCC is characterized by a highly immunosuppressive microenvironment, with elevated levels of Tregs, MDSCs, and immune checkpoint molecule expression. These features may enable ccRCC to evade immune responses, potentially counteracting the pro-immunogenic effects of ICD. The interaction between DAMPs released during ICD and the immune cells in the ccRCC TME could influence the balance between immune activation and suppression, shaping the overall treatment response. Investigating whether ccRCC-specific immune evasion mechanisms, such as alterations in antigen presentation or DAMP signaling pathways, attenuate ICD-mediated immune responses is a critical area for future exploration.

ICD often occurs in conjunction with or influences other regulated cell death (RCD) mechanisms, including pyroptosis, ferroptosis, and cuproptosis. These interactions play a crucial role in shaping the TME and influencing cancer progression. For instance, pyroptosis, a pro-inflammatory form of cell death, may synergize with ICD to amplify anti-tumor immune responses through the release of pro-inflammatory cytokines and DAMPs [28]. Ferroptosis, characterized by iron-dependent lipid peroxidation, has been shown to modulate immune responses by altering the TME, which could enhance or suppress ICD-related effects [29]. Similarly, cuproptosis, driven by copper-induced mitochondrial proteotoxicity, may influence cellular metabolism and immune signaling pathways relevant to ICD [30].

Compared to existing ICD-related prognostic models, our model was distinctively innovative. For instance, in the study by Zhou et al., they focused on predicting patients' survival and susceptibility to immunotherapy through ICD-associated gene signatures [31]. In contrast, our article specifically investigates the role of the FOXP3 gene, its direct implications on the immune environment, and its impact on tumor progression in ccRCC, providing a more targeted approach to understanding and treating this specific cancer type. This targeted approach allowed for more precise description and prediction of clinical outcomes in ccRCC patients, providing more specific and personalized treatment guidance compared to the broad ICD markers used by Brahmer et al. in lung cancer research, which, while correlated with prognosis, did not analyze the prognostic impact across different tumor subtypes [32]. Moreover, our study provided a detailed analysis of the expression patterns of FOXP3 in ccRCC and its specific impact on the tumor immune environment, discussing its potential as a therapeutic target. This contrasted with general ICD models that often overlook the complex roles of specific regulatory factors within the TME. For example, although Martins et al. identified multiple genes associated with tumor immunity, they did not delve into the unique roles of these genes in specific TMEs [33]. Our focused analysis not only enhances understanding of ccRCC treatment strategies but also offers the potential for tailored therapeutic approaches based on individual gene expression, thus improving treatment precision and effectiveness.

FOXP3 is a transcription factor with a dual role in cancer biology, functioning as an immune suppressor in certain tumors and as a direct regulator of tumor cell behavior in others. In ccRCC, our findings show that FOXP3 promotes an immunosuppressive TME by enhancing regulatory T cell infiltration and upregulating immune checkpoint molecules, leading to immune evasion. These effects align with its role in many other solid tumors, such as colorectal and breast cancer, where FOXP3 expression correlates with poorer prognosis due to its ability to inhibit anti-tumor immune responses [34]. In NSCLC, FOXP3 promotes tumor progression through the Wnt-β-catenin signaling pathway [35]. However, in hepatocellular carcinoma (HCC), FOXP3 is associated with improved outcomes, where it may act through distinct pathways, such as modulating TGF-β/Smad signaling [36]. The mechanistic contradictions in FOXP3’s role across cancer types likely stem from its interaction with cancer-specific signaling pathways and the distinct cellular context of each tumor. In ccRCC, FOXP3 appears to promote tumor progression not only by modulating the TME but also through direct effects on tumor cell proliferation and migration, as demonstrated in our functional experiments. Pathway enrichment analyses revealed that FOXP3 is associated with the activation of oncogenic pathways, including Wnt/β-catenin and TGF-β, which may contribute to its tumor-promoting effects. These findings suggested that FOXP3 serves as both a regulator of immune suppression and a mediator of tumor cell behavior in ccRCC, representing a convergence of its dual roles.

This study has several limitations that warrant discussion. Although we utilized multiple datasets (TCGA-KIRC, GSE29609, and GSE159115) for robust analysis, the sample size for some subgroup analyses was limited, potentially affecting the generalizability of our findings, which should be validated in larger, multicenter cohorts. Our experimental validation of FOXP3 knockdown effects was restricted to two ccRCC cell lines (Caki-1 and 769-P), limiting the representation of ccRCC tumor heterogeneity; future studies should incorporate additional cell lines and in vivo models for comprehensive validation. Additionally, the clinical utility of the ICDS model, such as guiding immunotherapy decisions, requires prospective validation in clinical trials to ensure feasibility and reliability in clinical settings.

## Conclusion

Our study underscores the critical role of ICD in ccRCC prognosis and the potential of targeting ICD pathways to improve treatment outcomes. FOXP3 knockdown experiments showed reduced cell proliferation and migration, highlighting its role in tumor progression. These findings suggest that targeting ICD pathways, particularly FOXP3, could improve ccRCC treatment strategies.

## Supplementary Information

Additional file1Genetic Subtyping Based on Differential Gene Expression in ccRCC. A: Consensus clustering further classifying ccRCC samples into geneclusters A and B based on differential gene expression; B: Heatmap displaying the gene expression patterns and clinical variables distribution across geneclusters; C: KM survival curves for patients stratified by geneclusters, indicating poorer prognosis for genecluster B; D: Differential expression of prognostic ICD-related genes between geneclusters

Additional file2Drug Sensitivity Analysis in ccRCC Stratified by ICDS. A-L: Assessment of IC50 of various chemotherapy drugs for renal cancer, comparing the suitability of drugs like Axitinib, Bosutinib, Gefitinib, and others across high and low ICDS groups

## Data Availability

The datasets analyzed in the current study are publicly available from The Cancer Genome Atlas (TCGA, https://www.cancer.gov/tcga) and the Gene Expression Omnibus (GEO, https://www.ncbi.nlm.nih.gov/geo/). Specific dataset accession numbers and processing details are provided in the manuscript or supplementary materials.
