# Supplementary figures and images for: FOXP3 as a prognostic marker and therapeutic target in immunogenic cell death modulation for clear cell renal cell carcinoma
Source: Discov Oncol. 2025 Jan 30;16:102. doi: 10.1007/s12672-025-01831-w (PMC11782763; doi:10.1007/s12672-025-01831-w)

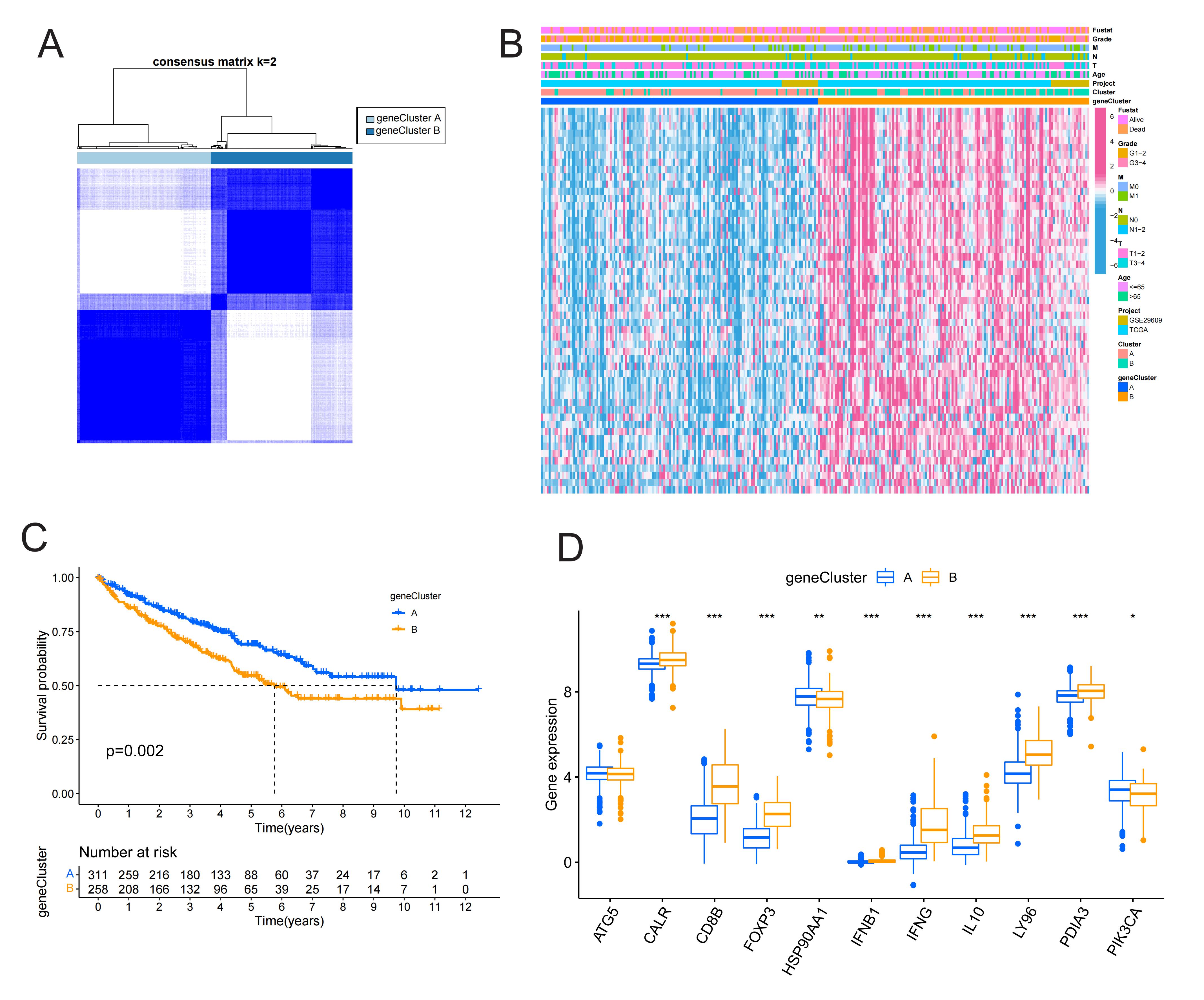

Supplement: Supplementary file 1 — Additional file1Genetic Subtyping Based on Differential Gene Expression in ccRCC. A: Consensus clustering further classifying ccRCC samples into geneclusters A and B based on differential gene expression; B: Heatmap displaying the gene expression patterns and clinical variables distribution across geneclusters; C: KM survival curves for patients stratified by geneclusters, indicating poorer prognosis for genecluster B; D: Differential expression of prognostic ICD-related genes between geneclusters [file 12672_2025_1831_MOESM1_ESM.jpg]

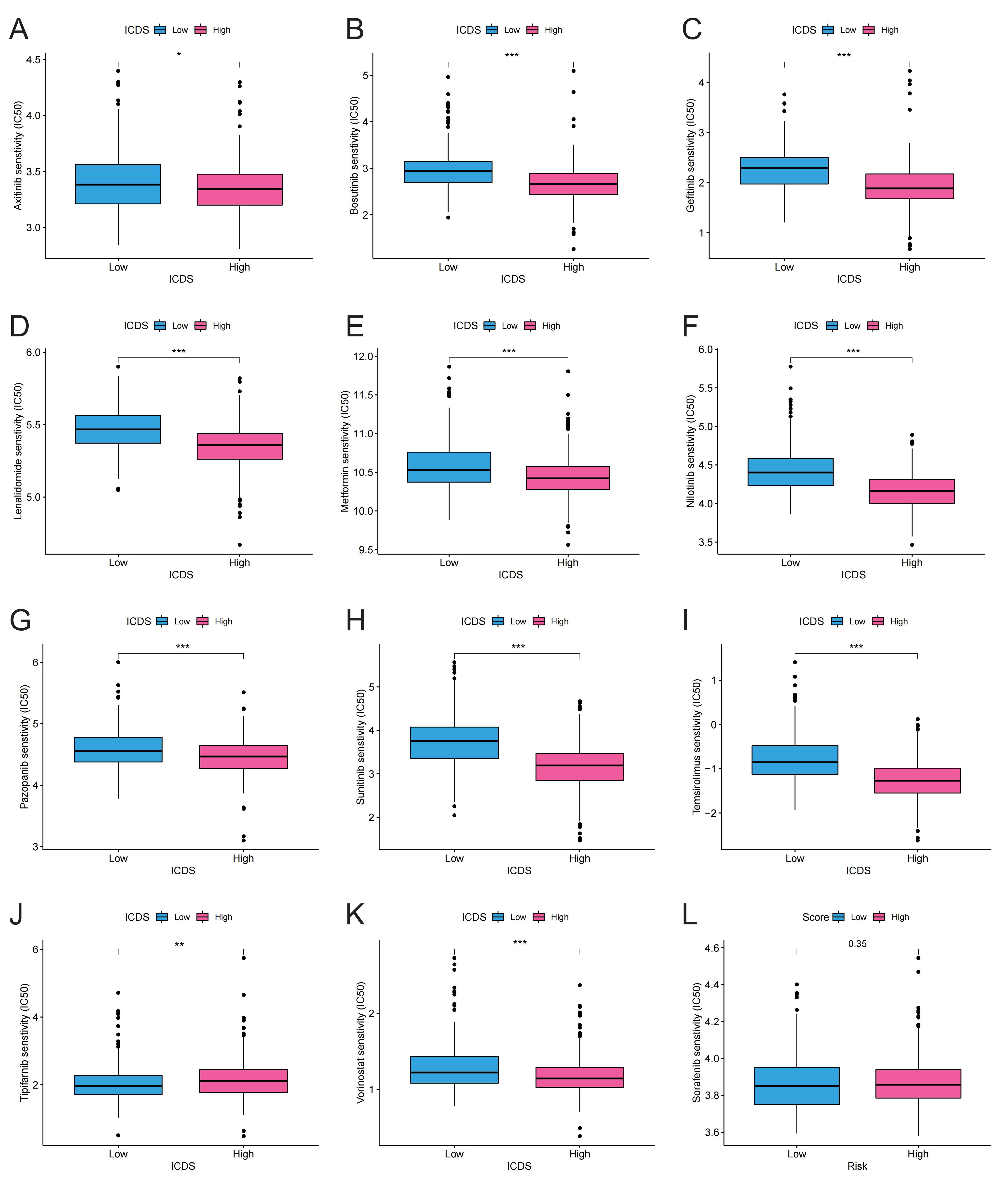

Supplement: Supplementary file 2 — Additional file2Drug Sensitivity Analysis in ccRCC Stratified by ICDS. A-L: Assessment of IC50 of various chemotherapy drugs for renal cancer, comparing the suitability of drugs like Axitinib, Bosutinib, Gefitinib, and others across high and low ICDS groups [file 12672_2025_1831_MOESM2_ESM.jpg]
